# Impact of Snow on Underground Smoldering Wildfire in Arctic-Boreal Peatlands

**DOI:** 10.1021/acs.est.4c08569

**Published:** 2025-02-06

**Authors:** Yunzhu Qin, Yichao Zhang, Yuying Chen, Shaorun Lin, Yang Shu, Yuhan Huang, Xinyan Huang, Mei Zhou

**Affiliations:** † Research Centre for Smart Urban Resilience and Firefighting, Department of Building Environment and Energy Engineering, The Hong Kong Polytechnic University, Kowloon 999077, Hong Kong SAR; ‡ School of Civil and Environmental Engineering, University of Technology Sydney, Sydney, NSW 2007, Australia; § College of Forestry, Inner Mongolia Agricultural University, Hohhot 010019, China; ∥ Department of Mechanical Engineering, University of California, Berkeley, Berkeley, California 94702-5800, United States

**Keywords:** overwintering fires, outdoor experiment, peat fire suppression, snow precipitation

## Abstract

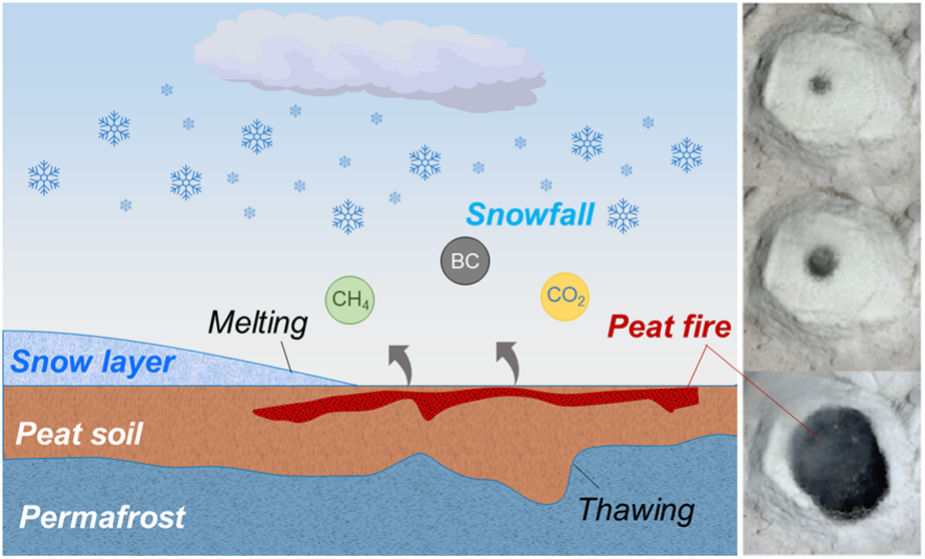

Overwintering peat fires are re-emerging in snow-covered Arctic-boreal regions, releasing unprecedented levels of carbon into the atmosphere and exacerbating climate change. Despite the critical role of fire–snow interactions in these processes, our understanding of them remains limited. Herein, we conducted small-scale outdoor experiments (20 × 20 × 20 cm^3^) at subzero temperatures (−5 ± 5 °C) to investigate the impact of natural snowfall and accumulated snow layers (up to 20 cm thick) on shallow smoldering peat fires. We found that even heavy natural snowfalls (a maximum water equivalent snowfall intensity of 1.1 mm/h or a 24 h accumulated snowfall water equivalent precipitation of 7.9 mm) cannot suppress a shallow smoldering peat fire. A thick snow cover on the peat surface can extract heat from the burning front underneath, and the minimum thickness of the snow layer to extinguish the peat fire was found to be 9 ± 1 cm at subzero temperatures, agreeing well with the theoretical analysis. Furthermore, larger-scale field demonstrations (1.5 × 1.5 m^2^) were conducted to validate the small-scale experimental phenomena. This work helps us to understand the interactions between fire and snow and reveals the persistence of smoldering wildfires under cold environments.

## Abstract 2

This study reveals the interaction between snowfall, snow layers, and overwintering peat fires, highlighting their contribution to carbon emissions and climate change in Arctic-boreal regions.

## Introduction

Peatlands are important ecosystems that have accumulated partially decomposed vegetation residues under acidic, water-saturated and anaerobic conditions.^1^ Although peatlands only cover ∼3% (4 × 10^6^ km^2^) of Earth’s land surface, they store over one-third of the global soil organic carbon (500–600 Gt C), approximately equal to those stored in living plants and atmosphere.^2−6^ The global peatlands are mainly distributed in tropical (primarily Southeast Asia) and Arctic-boreal regions (the northern high latitudes of the Americas, Europe, and Asia),^2,7^ playing an important role in promoting carbon cycling, regulating hydrological processes, and nurturing biodiversity.^7,8^

However, global peatlands are becoming more vulnerable to severe and frequent wildfires due to the accelerating climate change.^9−12^ Over the past few decades, the increasing prevalence of deep underground peat fires has led to the widespread destruction of peatland ecosystems and substantial emissions of greenhouse gases (GHGs).^13−16^ These GHG emissions, in turn, might give positive feedback to climate change, posing a severe threat to peatland ecosystems by increasing wildfire risk,^17−20^ carbon loss,^21−24^ permafrost thawing,^25−30^ and atmospheric pollution (e.g., CO, NO_*x*_, PM_2.5_, etc.).^14,31−34^ Furthermore, compared to the vegetation consumed by the surface fires, peatlands are not able to recover rapidly following a deep-propagated fire event, resulting in the irreversible release of carbon into the atmosphere.^10,35^ In Arctic-boreal regions, even though the cold environment and (frozen) soil water may restrict the severity of fires, recent measurements indicate that wildfires are erupting at a record-breaking pace.^10^ Many overwintering fires have been observed in Alaska and Northwest Territories, Canada, which may account for ∼3.5 Tg of carbon emissions in the last two decades.^36^

Smoldering is the dominant burning phenomenon of wildfires in peatlands.^20^ It is a persistent type of combustion that is characterized as a slow, low-temperature, and flameless process in porous charring fuels.^37−39^ Smoldering wildfires occur more readily than flaming fires and survive under lower temperatures, higher moisture contents (MCs), and lower oxygen concentrations.^40−44^ For example, our previous laboratory experiments have demonstrated that smoldering peat fires can survive below −40 °C^24^ and persistently burn 1 m below the ground for weeks.^45^ Furthermore, smoldering fire spots can creepingly spread underground for months and even years, awaiting the advent of dry and warm seasons to flare up, known as “overwintering fires.”^36,45−47^ Limited studies have explored the environmental influences from perspectives of topography change,^48^ hydrological regime,^49^ precipitation suppression,^50,51^ diurnal variation,^52^ etc. Nevertheless, complex smoldering fire behaviors in peatland are still poorly understood, requiring more fundamental research.

Snow is a crucial part of Arctic-boreal ecosystems that covers these regions for up to 9 months in a year,^53^ which plays important roles in land-surface energy balance.^54^ However, spring snow cover was found to decrease 7–11% per decade over the Northern Hemisphere since the 1970s, due to the accelerating climate change.^55^ The reduction in snow cover will expose darker surfaces like soil or vegetation with lower albedo, weakening the role of reflecting solar radiation back into space.^56^ As a result, these surfaces with lower reflectivity absorb more solar radiation, creating a positive feedback loop to amplify the effects of climate change. Apart from climate change, wildfires are another driver of the snow melting, which further accelerates and exacerbates the effect of climate change.^30,57^

Recently, more peat fires occurring in snow-covered areas have been observed and detected by remote sensing technology,^36^ including peat fires burnt under snow cover at −60 °C in *“the Pole of Cold”*, Russia. Although peat fires are recognized as a key contributor to the snow melting and permafrost thawing, whether the snowfall (SF) and snow cover will, in turn, affect the burning dynamics of smoldering wildfires is still unclear. Therefore, the objectives of this study are (1) to investigate the impact of natural snowfall (SF) on peat fires, specifically if a natural snowfall can suppress a peat fire; (2) to examine the role of accumulated snow layer (SL) on peat fires, considering whether it acts as a surface insulation layer or an extinguishing agent; and (3) to quantify the potential influence of snowmelt on the behaviors of peat fires. To fill these knowledge gaps, it is necessary to thoroughly investigate the interactions between snow and peat fires.

## Materials and Methods

### Peat Soil Samples

Typical Arctic-boreal moss peat soils from Estonia were selected for the experiments. This peat with uniform density and particle size can ensure better experimental reproducibility, as demonstrated in our previous studies.^45,58^ The peat soil had a porous structure (porosity ≈0.9), a high organic content (>95%), and a dry bulk density (ρ_p_) of 145 ± 15 kg/m^3^. Although natural peat moisture content (MC) fluctuates with seasonal changes, climate and water table levels,^59^ our tests oven-dried the peat to about 5% MC (dry mass basis)^24,40,60^ to eliminate the influence of pre-melting soil moisture.

### Peat Fire Tests

**Figure 1:**
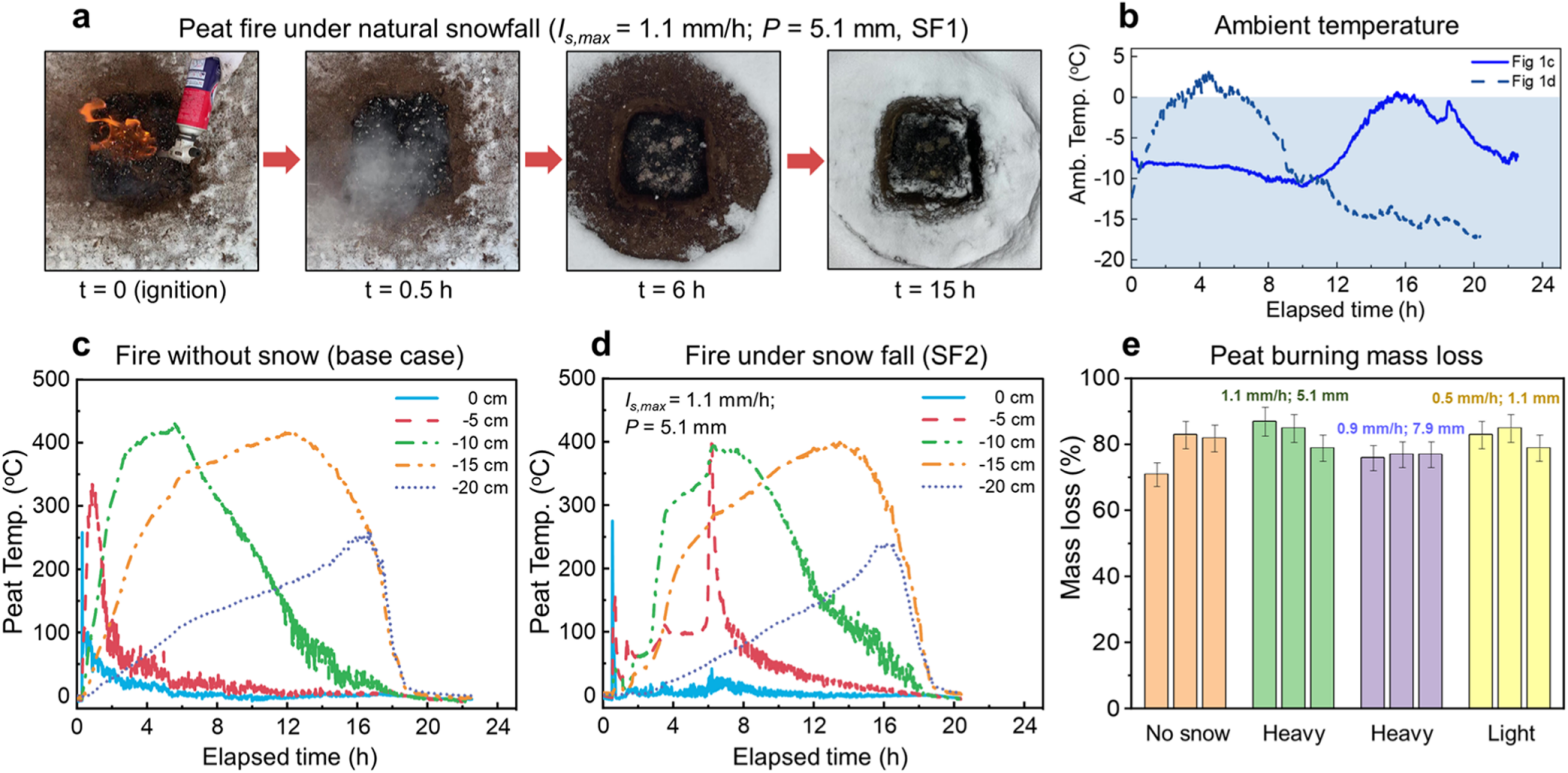
Peat fires under snow-free and natural snowfall conditions. (a) Snapshots of underground fire test under the heavy snowfall; see Video S1. Thermocouple measurements of (b) ambient temperatures during tests, with an average of subzero condition (−5 ± 5 °C), (c) burning peat without snowfall, and (d) peat fire under a heavy natural snowfall (*I*_s,max_ = 1.1 mm/h; water equivalent value of *P* = 5.1 mm). (e) Peat soil burning mass loss *vs.* snowfalls.

All experiments were performed outdoors in the boreal region of Inner Mongolia, China (Figure S1a), so they were more realistic than lab tests. The local diurnal temperature variation was lower than 20 °C with an average temperature of −5 °C (Figure 1). Small-scale fire test pits with dimensions of 20 × 20 × 20 cm^3^ were dig in the outdoor frozen soil layer to simulate the real fire scenarios. The surrounding frozen nonpeat soils had an MC of above 100%, which was wet enough to isolate the tested peat fire.

For each experiment, the peat sample was naturally placed in the pit without manual compaction. After at least 2 h of equilibration with the surrounding temperature, a propane flame was used to ignite the peat soil from the top surface for around 2 min. An array of five K-type thermocouples with a bead diameter of 1 mm was inserted into the axis of the peat at different depths (from 0 cm (surface) to −20 cm (bottom) at an interval of 5 cm) to measure the vertical temperature profile at a time interval of 1 min. Another thermocouple was placed near the ground to record the ambient temperature. For each fire scenario, at least three repeating tests were conducted to ensure the test reproducibility.

### Design of Snow Impact

There are two types of snow impacts: one is the dynamic snowfall, and the other is the snow layer accumulated from the previous snowfall, while both impacts can occur together. Therefore, three groups of field experiments were designed, as illustrated in Figure S1b, to investigate the snow impact on Arctic-boreal peat fires:

(I) Natural snowfall (SF) tests. The ignition and peat fire propagation processes were conducted with ongoing natural snowfall. Similar to the classification of rainfall intensity, the intensity of snowfall also has two classification standards, namely, the water equivalent maximum snowfall intensity and the cumulative water precipitation in 24 h. Measured by the ground meteorological station, they are divided into light, moderate, heavy, and violent, depending on its intensity and accumulation of its equivalent liquid water during a certain period,^61,62^ as shown and compared in Table 1. During the test, both snowfall intensity (water equivalent value, mm/h) and 24 h accumulated precipitation were confirmed by National Meteorological Science Data Centre (https://data.cma.cn/dataService, accessed Jan 15, 2025). To ensure the repeatability, three independent experiments were conducted at the same time under each natural snowfall scenario. The total mass loss before and after the fire was also measured to indicate the influence of snow on peat burning.
(II) Accumulated snow layers (SL) tests. Fresh snow layer samples were collected right after the natural snowfall. In the experiment, the peat fire was first ignited for the same 2 min without snow. Then, a snow layer with a given thickness (δ_SL_) from 1 to 20 cm was placed on the top surface. Snow thickness was controlled and calculated using snow weight and average bulk density to minimize measurement errors. The bulk density of fresh snow layer was measure as ρ_SL_ = 265 ± 20 kg/m^3^. Postfire soil residues were collected to determine the burning mass loss and the residual moisture content. The average environmental temperature during the whole experimental period was around −5 °C, and the minimum temperature was around −18.2 °C, as summarized in Table 2.
(III) Large-scale demonstrations with both SF and SL. The experimental burn area was designed to be 1.5 m × 1.5 m on frozen soil with a depth ranging from 15 to 20 cm. The ignition area of 15 cm × 15 cm was positioned at the corner and heated by a 2 min flame. The fire was initiated during a moderate natural snowfall (*I*_s,max_ = 0.7 mm/h; *P* = 2.6 mm, water equivalent value), while natural snow accumulation occurred in regions where the peat fire had not yet spread. Therefore, both the effects of natural snowfall and accumulated snow layer could be observed and analyzed in this demonstration.

**Table 1:** Classification of Snowfall Intensity, by Using the Liquid Water Equivalent Systems (LWES), Compared with Rainfall in Brackets^61,62^

classification	snowfall (rainfall) intensity (mm/h)	snowfall (rainfall) in 24 h (mm)
light	<1 (<2.5)	<2.5 (<10)
moderate	1–2.5 (2.5–10)	2.5–5 (10–25)
heavy	2.5–10 (10–50)	5–10 (25–50)
violent	>10 (>50)	>10 (>50)

**Table 2:** Summary of Experimental Conditions^a^

test no.	*T̅*_∞_ (°C)	*T*_∞,min_ (°C)	*I*_s,max_ (mm/h)	*P* (mm)	δ_SL_ (cm)	*m*_SL_^′′^ (kg/m^2^)	fire (Y/N)
base case (no snow)	–8.7	–10	0	0	0	0	Y
SF1–SF3	–8.5	–17.3	1.1	5.1 (heavy)	N.A.	N.A.	Y
SF4–SF6	–2.6	–6.7	0.9	7.9 (heavy)	N.A.	N.A.	Y
SF7–SF9	–3.8	–6.7	0.5	1.1 (light)	N.A.	N.A.	Y
SF10 (large demo)	–5.5	–11	0.7	2.6 (moderate)	N.A.	N.A.	Y
SL1	–11.7	–18.2	0	0	3	8.0	Y
SL2	–3.5	–12.9	0	0	3	8.3	Y
SL3	–4.1	–12.6	0	0	4	12	Y
SL4	–0.9	–8.7	0	0	6	16	Y
SL5	–5.1	–9.5	0	0	7	18	Y
SL6	–1	–8.9	0	0	8	20	N
SL7	–8.7	–10	0	0	8.5	22	N
SL8	–6.6	–10.9	0	0	10	26	N
SL9	–5.1	–9.5	0	0	10	27	N
SL10	–6.8	–8.1	0	0	10	25	N
SL11	–6.5	–11	0	0	12	30	N
SL12	–7.1	–14.1	0	0	15	42	N
SL13	–6.9	–12.4	0	0	17	48	N
SL14	–4.8	–9.5	0	0	20	52	N

a Average (*T̅*_∞_) and minimum ambient temperature (*T*_∞,min_), maximum natural snowfall intensity (*I*_s,max_), 24 h accumulated snowfall precipitation (*P*), snow layer thickness (δ_SL_), and area density of the snow layer (*m*_SL_^*′′*^). Note that snowfall intensity uses water equivalent value

## Results and Discussion

### Underground Peat Fire Phenomena

#### Fire without Snow (Base Case)

Our previous laboratory experiments in freezer have revealed that the fire threshold of dry peat could be lower than −45 °C, when the peat was dried.^24^ Herein, we first validated the peat fire behaviors in real soil land under subzero field conditions (−5 ± 5 °C). Figure 1c describes a temperature evolution of a baseline experiment without snow under a mean ambient temperature of −8.7 °C (a snapshot is shown in Figure S2). Once ignited from the top surface, the smoldering fire successfully propagated downward to the bottom of the peat layer in around 16 h. After fire, a thin black char layer was observed on the top free surface that was not burnt completely into the white ash because of a larger environmental cooling.^63^ The top residual char-and-ash layer acts as an insulation to help maintain a high smoldering temperature beneath (e.g., ∼ 300 °C at −5 cm vs ∼400 °C at −15 cm). Afterward, near the end of the fire spread, the measured temperature near the bottom was about 200 °C where the smoldering front could no longer propagate downward, leaving the other black char layer. At about 20 h, the underground smoldering fire burnt out, and a mixture of unburnt chars, ashes, and undisturbed peat was observed in the pit.

#### Fire with a Natural Snowfall (SF)

Figure 1d shows the thermocouple measurements of a peat fire under heavy natural snowfall (*I*_s,max_ = 1.1 mm/h; *P* = 5.1 mm, water equivalent value), and the corresponding burning process is avaiable in the Video S1. In general, compared with Figure 1c, the trend of fire propagation was only slightly influenced by the snowfall, except for the fire near the top surface. Therefore, such a snowfall was not able to suppress the smoldering peat fires. To be specific, when the snow reached the burning area, the temperature near the top surface (−5 cm) decreased and fluctuated, because the top peat layer was wetted, and the fire was partially and temporarily extinguished.

However, due to the strong evaporation and the water absorption in the upper soil layer, it was difficult for the melting snow to penetrate and arrive at the deeper soil layer. Therefore, the temperatures below the top layer (e.g., −10 and −15 cm) quickly increased to about 400 °C, just like the base case in Figure 1c. As the fire grew, the top peat layer was dried and burnt as well, and the entire peat sample was burnt out eventually. Figure 1e further compares the burning mass losses of three repeating tests under heavy and light snowfalls to those without snowfall, with a 5% error bar representing potential systematic errors in the measurement process. The burning mass loss fluctuates around 80% in all scenarios, showing a negligible difference. This further demonstrates that a snowfall of *I*_s,max_ = 1.1 mm/h or *P* = 7.9 mm (water equivalent value) cannot effectively extinguish the underground smoldering peat fire. This supports many field observations available in the Arctic-boreal peatland, where people see that the underground smoldering peat fires continue to burn under a snowfall.

#### Fire with Accumulated Snow Layers (SL)

**Figure 2:**
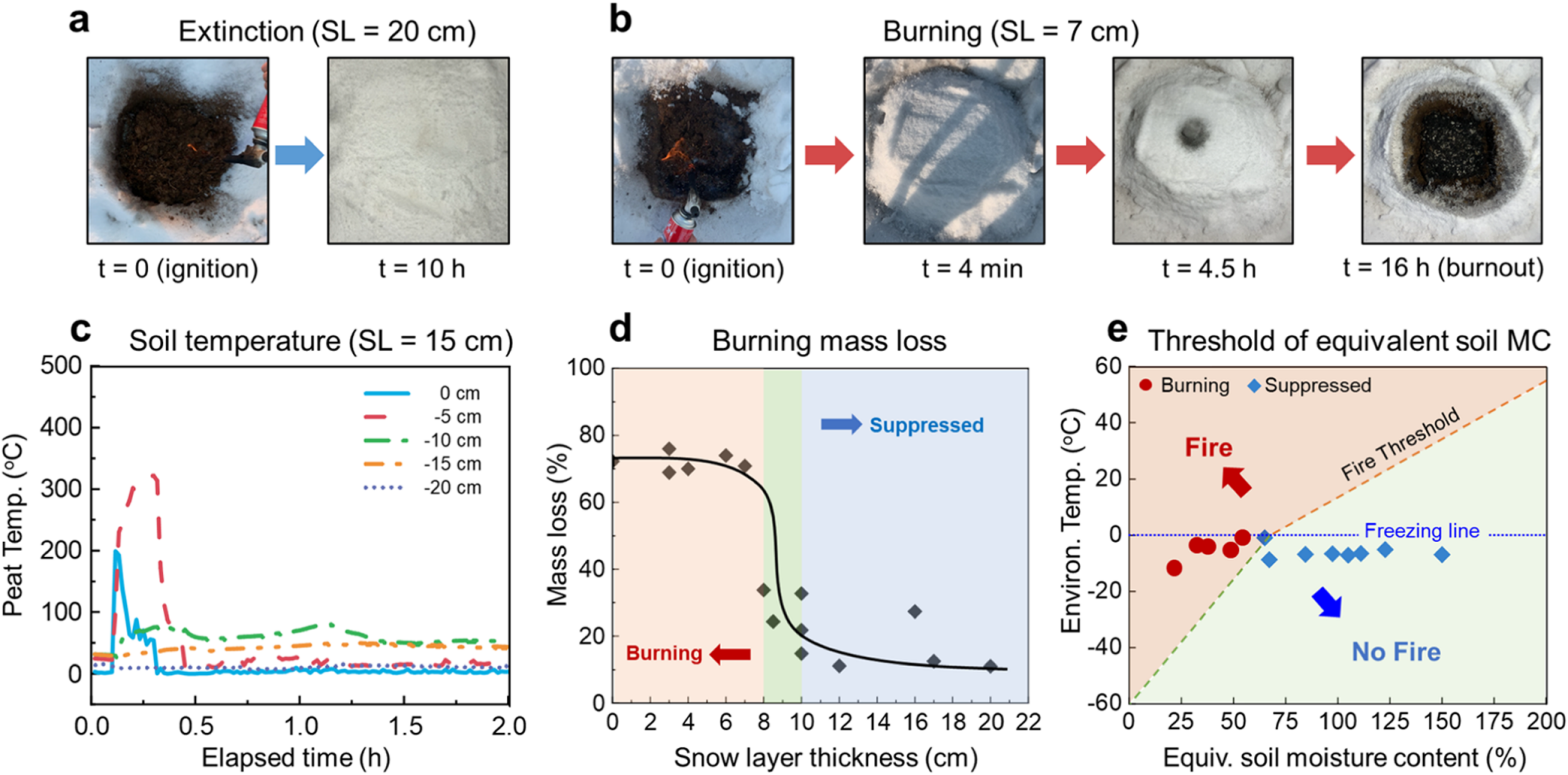
Peat fires under varying snow layer thicknesses. (a, b) Snapshots of a successful fire suppression under snow layer (δ_SL_ = 20 ± 1 cm), and a case of burning under snow layer (δ_SL_ = 7 ± 0.5 cm). The snow layer thickness δ_SL_ is calculated by the snow weight and average bulk density. The original video: Videos S2 and S3. (c) Thermocouple measurements of fire extinction under a snow layer of 15 cm. (d) Mass loss of (dried) peat soil under different snow layer thicknesses, where the mean environmental temperature is around −5 ± 5 °C. (e) Experimental data as a function of environmental temperature and equivalent soil moisture content, which agreed well with the curve of fire threshold obtained from previous lab tests.^24^

Figure 2a,b compares the successful fire suppression under a 20 ± 1 cm thick snow layer (estimated by snow weight and average bulk density) and the failed suppression under a snow layer of 7 ± 0.5 cm. Clearly, when the snow layer is thick enough, underground peat fire will be extinguished. Figure 2c further shows the temperature evolution of a peat soil in a successful fire-suppression case under a 15 cm thick snow layer. First, the temperature at −5 cm increased to above 300 °C so that the fire was successfully ignited to sustain smoldering propagation. Shortly after, its temperature significantly decreased to ambient temperature, while the fire front no longer propagated downward. The temperature measurements were continued for another 24 h, and no reignition was observed. Moreover, we observed some unmelted snow remained on the ground, whose thickness was found to increase with the initial snow layer thickness. On the other hand, if the snow layer thickness was reduced, it eventually became too thin to extinguish the fire. Thus, we can identify the threshold of snow layer thickness to suppress a peat fire.

### Threshold of Peat Fire under Snow Layers

Figure 2d summarizes soil burning mass losses below different snow layer thicknesses. Clearly, there is a minimum snow layer thickness of 9 ± 1 cm (or 23 ± 3 kg/m^2^) to suppress a smoldering underground fire at a mean environment temperature of −5 ± 5 °C. If the thickness of the snow layer was smaller than 8 cm, the burning mass loss of peat remains relatively stable at ∼75% (close to the no-snow case). After an intense smoldering fire, the moisture content of residue remained below 20%.

If the snow layer was thinner than 8 cm, the burning mass loss dropped sharply to 10–35%, which was mainly caused by the ignition process. For these cases, the (partially) melted thin snow layer increased the moisture content of originally dried peat to above 60%, and detailed data are summarized in Figure S3. Thus, the effect of the surface snow layer on suppressing the peat fire is similar to an increase in soil moisture content. For simplicity, we assume that the melting snow increases the soil moisture content uniformly to
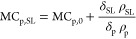
1where δ denotes the thickness of layer and the subscript “p” and “SL” represent the peat and snow, respectively. For example, the melting of a 10 cm thick snow cover above a 20 cm thick dry peat (MC_p,0_ ≈ 5%) will increase soil MC to 96%.

Based on this analogy, Figure 2e summarizes the experimental relationship between the environmental temperature and the equivalent peat moisture content, where the smoldering fire threshold (“fire” and “no fire” zones) found previously^24^ was included for analysis. For thin snow layers (<8 cm) that were not able to suppress the peat fires, all of these burning cases are exactly located in the fire zone (see Table 2). For thick snow layers (>8 cm), all extinguished cases are located in the no fire zone. In other words, the effect of the snow layer on suppressing peat fire can be explained by an increased equivalent soil moisture content and the smoldering fire threshold which may be used to evaluate the underground fire risk in the Arctic-boreal regions.

### Fire-Suppression Limit of Equivalent Precipitation

For the peat fire below the snow layer, the hot fire emissions can gradually melt the snow into liquid water that penetrates and cools the soil to suppress the peat fire (if the snow cover is thick enough). By considering the melting time of the snow layer, the fire-suppression effect is equivalent to the snowfall (or rainfall). For example, if a snow layer of 480 g weight (equivalent water of 12 mm within the area of pit) is melted by peat fire in 2 h, its equivalent snowfall intensity is 6 mm/h. Then, we can obtain the equivalent snowfall intensity and equivalent liquid water depth for all snow layer tests.

**Figure 3:**
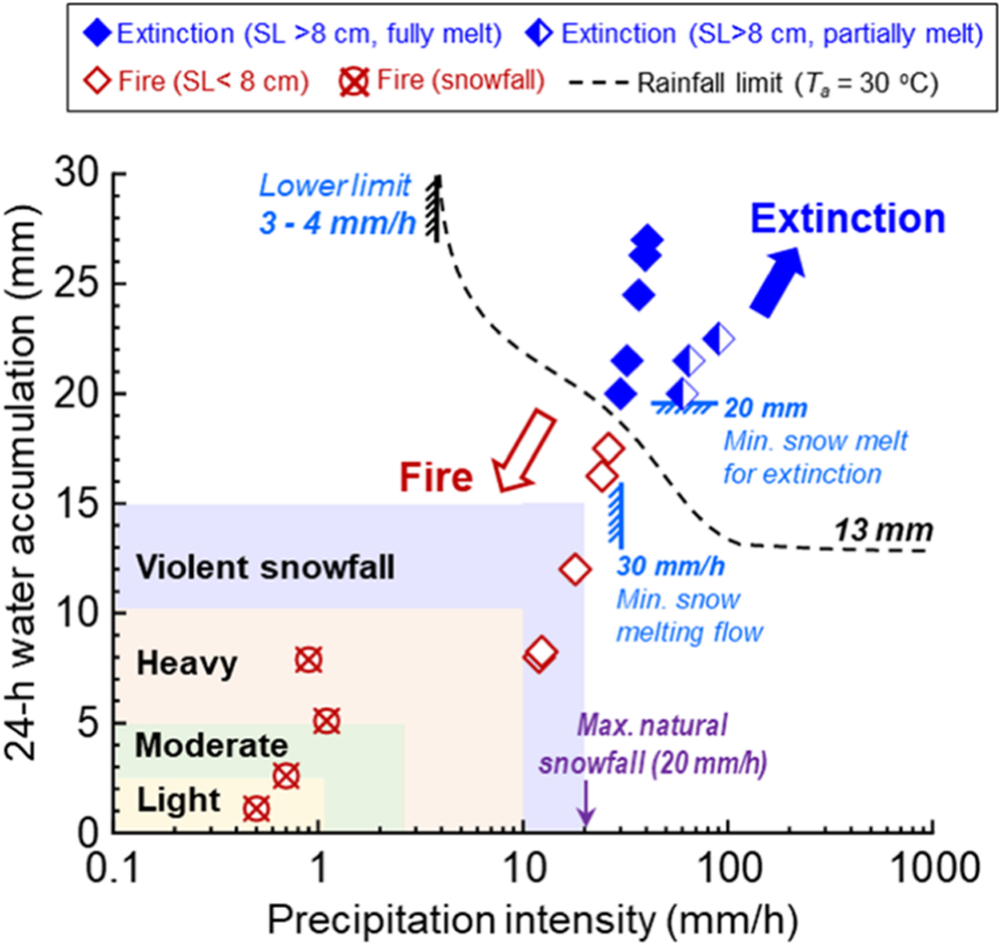
Peat fire-suppression limit of water equivalent snow at −5 ± 5 °C. Quadrilateral-shaped points represent equivalent melting water intensities in SL tests. Hollow red points represent SL tests without peat fire extinction. The solid (or half-hollow) blue points indicate peat fire extinction with entire (or partial) SL melted. The SL-suppression limit matches the limit of rainfall-suppression limit (dashed line)^51^ at 30 °C.

Figure 3 summarizes the equivalent liquid water depth within 24 h and precipitation intensity for all snowfall (circle markers) and snow layer tests (quadrilateral markers). First, the melting rate of snow increases with the thickness of the snow layer because a thicker snow layer can absorb the heat of fire emission more efficiently. Nevertheless, by further increasing the snow layer above 15 cm, it can extinguish the underground peat fire before it is fully melted so that its equivalent snowfall intensity starts to decrease. Then, there is a maximum equivalent snowfall intensity for the snow layer, which is found to be about 90 mm/h (water equivalent value) at a testing ambient temperature of −5 ± 5 °C (SL14 in Table 2). Note that this maximum value changes with the ambient temperature, and it is much larger than the historical maximum natural snowfall of around 20 mm/h (water equivalent value).^64^

Although the limited numbers of snowfall and snow layer test data cannot conclude a full fire-threshold curve, the resulting limiting curve should follow a trend similar to that for rainfall (Figure 3). By referring to the rainfall limit previously measured in a 30 °C lab environment,^51^ we can define a similar fire-threshold curve for snowfall in Figure 3. This snowfall limit should include both precipitation intensity and the total water amount caused by natural snowfall or accumulated snow layers. Specifically, the intensity should reach 30 mm/h (water equivalent value), and water accumulation should achieve 20 mm to effectively suppress a peat fire under −5 ± 5 °C. This extinction limit also follows the similar trend with previous rainfall-suppression experiments from ref (51) (dashed line in Figure 3).

### Theoretical Analysis

#### Minimum Snow Layer Thickness

**Figure 4:**
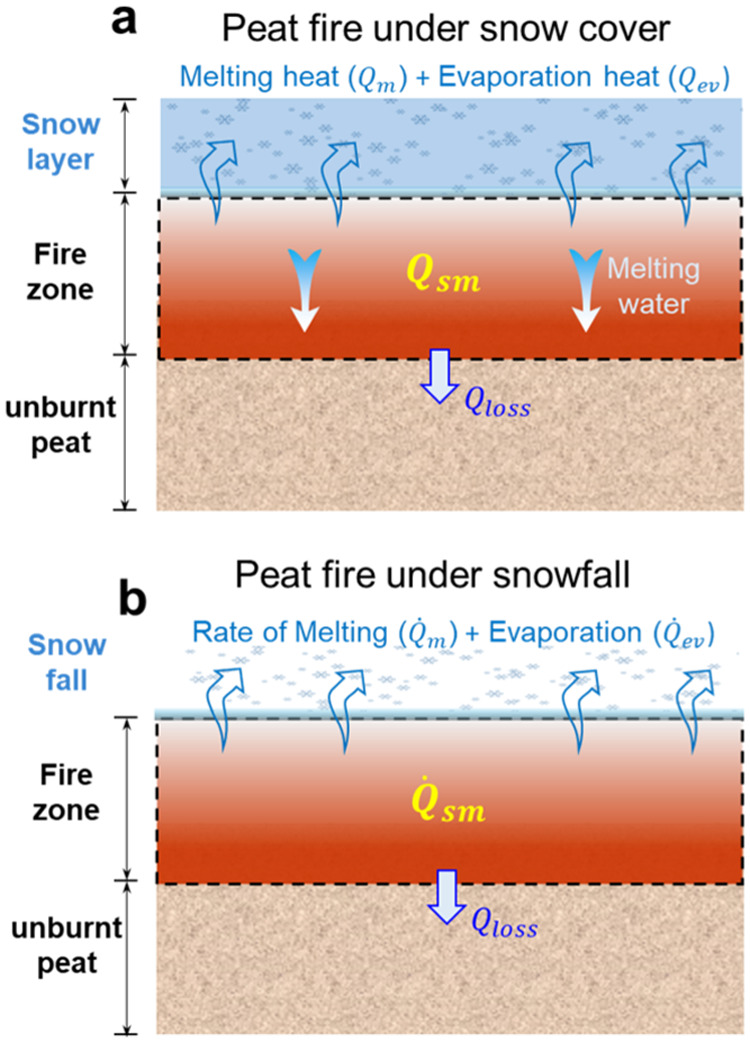
Illustrations of snow-fire interaction: (a) peat fire with a minimum snow layer and (b) snowfall where all the snow is melted and evaporated directly on the surface by the hot burning zone.

To physically explain the suppression mechanism and limit of peat fires by snow covers, the energy balance between the accumulated snow layers and smoldering fires is simplified in Figure 4a. When there is a snow layer above the underground peat fire, it will melt into water rapidly by the hot surface and the floating hot smoke from smoldering burning, so the meltwater will penetrate downward and cool down the underground burning zone. As the snow layer thickness increases, eventually the total heat released from underground peat fire can no longer overcome the cooling from the snow cover.

Then, the simplified energy-conservation equation can be established among heat released from the current smoldering fire zone (*Q*_sm_), energy storage in the preheated soil (*Q*_T_), heat absorption by snow melting (*Q*_m_), the evaporation of meltwater (*Q*_ev_), and other heat losses (*Q*_loss_) in eq 2 as
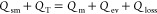
2

Since *Q*_T_ ≪ *Q*_sm_, it can be further specified as eq 3:
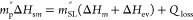
3where *m*_p_^′′^ = δ_sm_ρ_p_ is the burning mass of peat fire per area [kg/m^2^], δ_sm_ ≈ 3 cm is the thickness of the underground smoldering fire front,^45^ ρ_p_ is the density of dry peat, and Δ*H*_sm_ is the heat of smoldering combustion. For the snow layer, *m*_SL_^′′^ = δ_SL_ρ_SL_ is the weight of the accumulated snow layer per unit area, Δ*H* is the heat of snow melting, and Δ*H*_ev_ is the overall heat of water evaporation.

By further rearranging eq 3, the required minimum mass of the snow layer per area for fire suppression could be calculated as
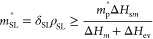
4where the environmental heat loss and the melting water penetrating through the fire region are neglected. The burning flux is estimated to be *m*_p_^′′^ = 4.0 ± 0.5 kg/m^2^ in this work. Key parameters can be found in the literature, where ρ_p_ = 145 kg/m^3^, *c*_p_ = 2 kJ/(kg K), Δ*H*_sm_ = 16 ± 4 MJ/kg,^65^ Δ*H*_m_ = 0.3 MJ/kg, and Δ*H*_ev_ = 2.7 MJ/kg (evaporate at 100 °C). By neglecting other energy losses, the minimum mass of snow per area to suppress the smoldering peat fire can be calculated as *m*_SL_^′′^ ≈ 20 kg/m^2^. As the bulk density of the snow layer was measured to be ρ_SL_ = 265 kg/m^3^, the minimum snow layer depth can be calculated as
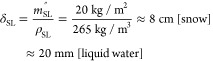
5which agrees well with the experiment observation of about 8 cm of snow layer (see Figure 2d) and the equivalent minimum liquid water depth of about 20 mm in Figure 3.

#### Minimum Snowfall Intensity

In the case of snowfall, the impact of snowfall precipitation is dynamic and different from that of the accumulated snow cover. To suppress the fire, the cooling rate of snow melting, and the subsequent water evaporation should be larger than the heat release rate from the smoldering front (see Figure 4b). Then, we can use the time derivative of eq 3 and introduce the precipitation intensity (*I* = *d*/Δ*t*)
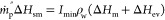
6where *ṁ*_p_^′′^ is the smoldering burning flux (burning mass loss rate per unit area) of peat soil. Therefore, the minimum (liquid water equivalent) snowfall intensity at a specific ambient temperature (*I*_min_) is
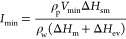
7where *V*_min_ = *ṁ*_p_^′′^/ρ_p_ is the minimum smoldering fire spread rate, which was measured to be 0.5 ± 0.1 cm/h,^58^ and ρ_w_ = 1000 kg/m^3^ is the density of water. Then, the minimum snowfall intensity is calculated to be 4 ± 1 mm/h (water equivalent value; see Figure 3). This explains why even a long-lasting heavy snowfall in this experiment (*I*_s,max_ = 1.1 mm/h) still cannot suppress a peat fire. Because the heat of melting snow is much smaller than the heat of evaporation, the minimum snowfall intensity for suppressing peat fire should be comparable to that of rainfall. Therefore, the natural snowfall needs to be very intense over a period to have the potential to extinguish a peat fire.

### Large-Scale Demonstrations

**Figure 5:**
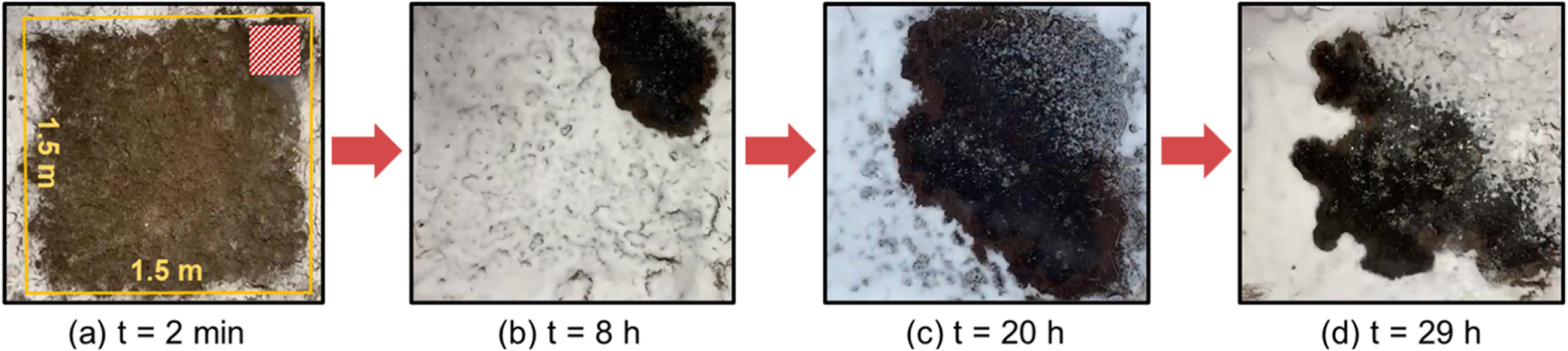
Key fire phenomena of the large demonstration under natural snowfall (water equivalent of *I*_s,max_ = 0.7 mm/h; *P* = 2.6 mm). The entire burning process lasts 40 h from ignition to burnout. (a) Spot ignition at the corner, (b, c) fire spreading with natural snow accumulation, and (d) finger-like spread caused by quenching on thin peat layers. The full video is available in Video S4.

Scaling up the small-scale fire tests to a larger field test is important to understand the real wildfire process. Figure 5 and Video S4 show the large peat fire demonstrations under moderate natural snowfall (*I*_s,max_ = 0.7 mm/h; *P* = 2.6 mm, water equivalent). After ignition at the corner, the peat fire started to spread outward in a fan-shaped pattern, while natural snowfall started to accumulate in the undisturbed areas (Figure 5a,b). After 20 h, fire still existed and the burning area expanded, confirming that this peat fire was not extinguished by this natural snowfall. This agrees well with the experimental observations in small-scale tests. Meanwhile, snow accumulated on the surface of the trailing edge of the fire front that had been burned out (Figure 5c).

Afterward, the leading edge of the fire front began to break up into separated fronts without consuming all the fuel in a finger-like manner (Figure 5d). A possible reason is that the peat layer at these locations is relatively shallow (measured as ∼5 cm) which cannot generate enough heat to overcome the heat loss caused by the snow.^48^ The entire burning process lasts 40 h from ignition to burnout. This large-scale experiment provided more information about the progression of smoldering peat fires under natural snowfall and accumulated snow layers. More and larger-scale field experiments under different environmental conditions (e.g., ambient temperature, wind, moisture content, snowfall, and topography) are needed to unravel the complex relationship between fire and snow in real peatlands.

## Environmental Implications

The Arctic environment plays a significant role in regulating global climate but has experienced warming at a rate greater than the global average (i.e., Arctic amplification^66^). This makes the region particularly vulnerable due to its heightened sensitivity to temperature changes. This vulnerability has been further exacerbated by increasing fire hazards in the Arctic-boreal peatlands. Herein, we further estimate the extent of vulnerable and safe peatland regions using the snowmelt extinction threshold identified in this work (minimum snow layer thickness of 8 cm at −5 ± 5 °C). ERA5-Land historical data and climate projections under two Shared Socioeconomic Pathways (SSP) from the Scenario Model Intercomparison Project for Coupled Model Intercomparison Project 6 (CMIP6) were used: an optimistic scenario (SSP1-2.6) and a pessimistic scenario (SSP5-8.5). The results indicate that from 1951 to 2020, decade-averaged fire-safe peatlands due to snowmelt have been declining (*p* = 0.0135). Furthermore, although the total area of peatlands with snow cover shows little variation, the area with thick snow (>8 cm) is projected to decline substantially (*p* < 0.001), decreasing by 11.5–54.3% by the end of the century under SSP1-2.6 (sustainability-focused, 150,365 km^2^) or SSP5-8.5 (fossil-fuel-reliant, 711,628 km^2^). These findings, detailed in the Supporting Information, highlight the increasing vulnerability of Arctic-boreal regions to peat fires.

The growing susceptibility of Arctic-boreal peatlands to fires has significant environmental consequences. Substantial emissions of greenhouse gases (GHGs) and aerosols, including black carbon (BC) and organic carbon (OC), can enhance Arctic amplification by increasing radiative forcing and trapping outgoing longwave radiation.^67^ Moreover, the deposition of BC on snow layers reduce surface reflectance through the snow-albedo feedback, accelerating snowmelt and temperature rise.^68^ Melting of snow and ice expose underlying low-albedo vegetation and soil,^56^ further increasing land solar radiation absorption and intensifying the Arctic amplification effect. Meanwhile, the increased exposure of peat and vegetation, combined with temperature-driven evaporation of fuel moisture, elevates wildfire risks in the Arctic-boreal region.

Arctic amplification and increased peatland vulnerability to fire also destabilize permafrost, leading to thaw, thermokarst formation, and ground subsidence.^22,29,69^ Permafrost thawing have expanded the active layer over the last 30 years,^70^ releasing significant soil carbon into atmosphere as GHG emissions such as CO_2_ and CH_4_.^71,72^ This not only shift Arctic-boreal peatland from net sink to net source of warming^27^ but also affects soil structure and hydrological systems.^30,73^ More importantly, meltwater mobilizes dissolved organic carbon (DOC) and burnt residues, such as fluoride, sulfate, and polyaromatic compounds, into the aquatic ecosystems,^31,74−77^ potentially altering nutrient cycling, shifting microbial community composition, and degrading water quality.

Thus, the increasing vulnerability of northern peatlands to fire, compounded with Arctic amplification, results in a feedback loop of intensified greenhouse forcing, permafrost thawing, and groundwater pollution. This emphasizes the urgent need for further research and strategies aimed at protecting Arctic-boreal ecosystems in the face of climate change.

## Supporting Information Available

Peat fire under heavy natural snowfall (Video S1) (MP4)
Fire suppressed by accumulated snow layer (Video S2) (MP4)
Pear fire with an accumulated snow layer (Video S3) (MP4)
The large peat fire demonstrations under moderate natural snowfall (Video S4) (MP4)
Overview of test area; illustrations of experimental design; snapshot of a base case of peat fire; moisture content of burnt residue; historical and projected peatland area with snow cover (PDF)
