# Supplementary material for: Impact of Snow on Underground Smoldering Wildfire in Arctic-Boreal Peatlands
Source: Environ Sci Technol. 2025 Feb 6;59(8):3915–24. doi: 10.1021/acs.est.4c08569 (PMC11883812; doi:10.1021/acs.est.4c08569)
Supplement: Supplementary file 5 — es4c08569_si_005.pdf [file es4c08569_si_005.pdf]

Supporting Information for:

Impact of Snow on Underground Smouldering Wildfire in Arctic-Boreal Peatlands

Yunzhu Qin [a,b,#], Yichao Zhang [a,#], Yuying Chen [a], Shaorun Lin [d,*], Yang Shu [c,*], Yuhan Huang [b], Xinyan Huang [a,*], Mei Zhou [c]

[a]*Research Centre for Smart Urban Resilience and Firefighting, Department of Building Environment and Energy Engineering, The Hong Kong Polytechnic University, Kowloon, 999077 Hong Kong SAR*

[b]*School of Civil and Environmental Engineering, University of Technology Sydney, Sydney, NSW, 2007, Australia*

[c]*College of Forestry, Inner Mongolia Agricultural University, Hohhot 010019, China*

[d]*Department of Mechanical Engineering, University of California, Berkeley, Berkeley, California 94702-5800, United States*

**Corresponding authors: xy.huang@polyu.edu.hk (X.H), shaorun.lin@berkeley.edu (S.L), and shuyang2018@imau.edu.cn (Y.S).*

[#]*Joint first authors: these authors contributed equally.*

This Supporting Information includes four figures:

Fig. S1. (a) Photo of the test area in this work in Inner Mongolia, China, and (b) diagram of experimental design: (I) the effect of natural snowfall, (II) the effect of accumulated snow layer on peat fires, and (III) large demonstration with both natural snowfall and accumulated snow layer.

Fig. S2. Fire phenomena of base case (without snow).

Fig. S3. Moisture content of burnt residue after smouldering peat fire with different snow layer thicknesses on the top surface.

Fig. S4. Historical and projected decade-averaged areas of peatland with snow cover, and those with snow layer thickness > 8 cm.

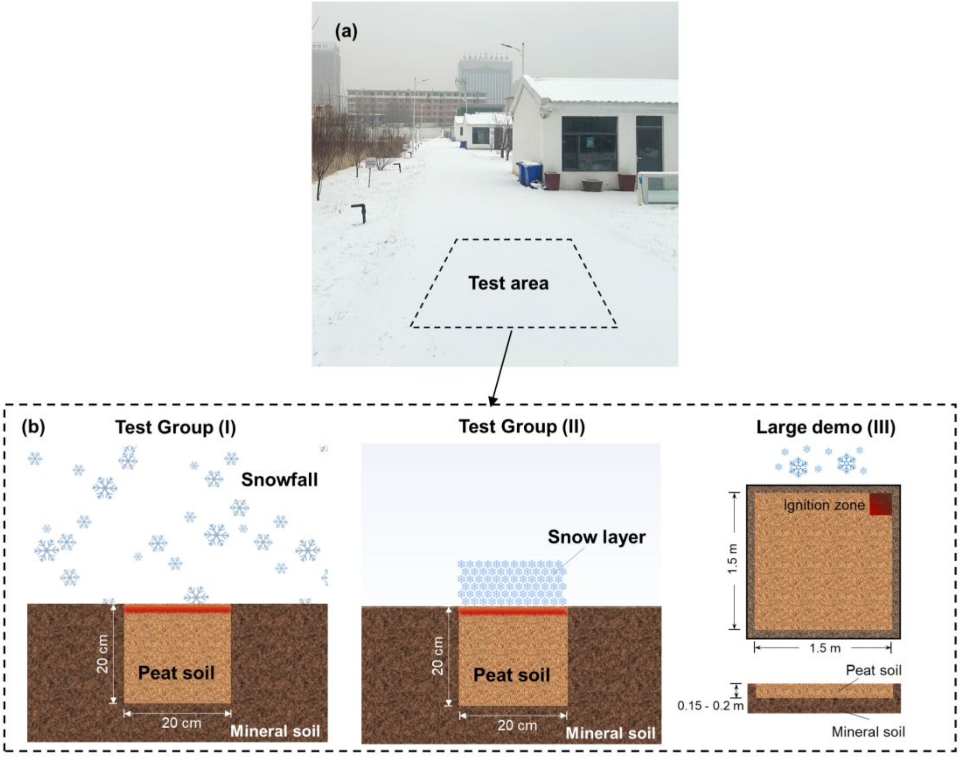

Fig. S1. (a) Photo of the test area in this work in Inner Mongolia, China, and (b) diagram of experimental design: (I) the effect of natural snowfall, (II) the effect of accumulated snow layer on peat fires, and (III) large demonstration with both natural snowfall and accumulated snow layer.

The outdoor experiments were conducted in Inner Mongolia, China (Fig. S1a). The average ambient temperature was around -5 ± 5 ^{o}C. Schematic diagrams of two groups of experiments are shown in Fig. S1b.

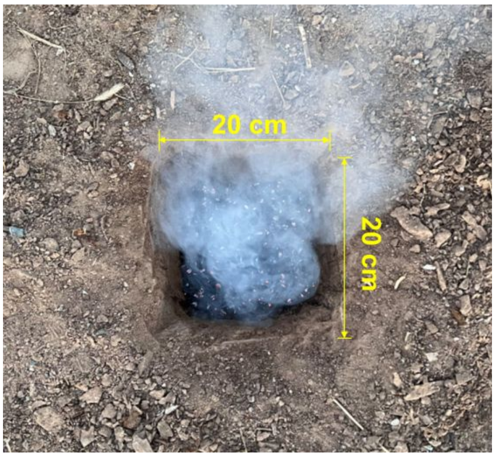

Fig. S2. Peat fire phenomena of base case without natural snowfall or snow layers.

Fig. S2 shows an example of small-scale peat fire test without snow. The surrounding mineral soil was not lined to maintain natural oxygen supply and heat loss conditions.

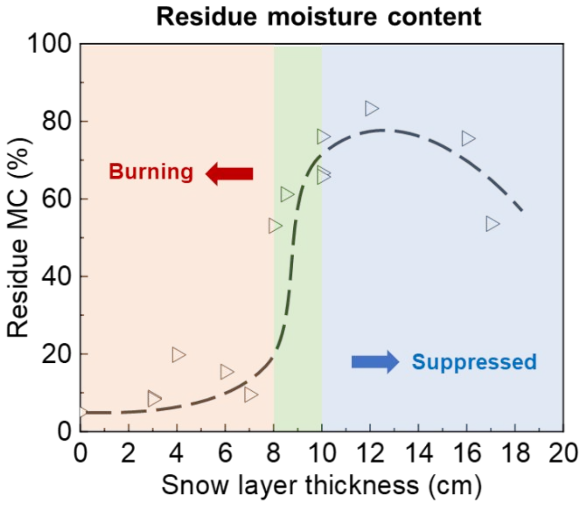

Fig. S3. Moisture content of burnt residue after smouldering peat fire with different snow layer thicknesses on the top surface.

Fig. S3 compares the moisture content (MC) of residue after burning under different thicknesses of snow layers. A clear boundary is found around 9 ± 1 cm. Below this thickness, the meltwater from snow is insufficient to extinguish the peat fire, resulting in residue moisture content remaining below 20%. Above this threshold, the residue MC increases to approximately 60%, corresponding to thermocouple data and experimental observations where the fire is successfully extinguished by the snow. When the snow layer thickness exceeds 15 cm, some snow remains unmelted after extinguishing the peat fire, leading to a slight decrease in residue MC.

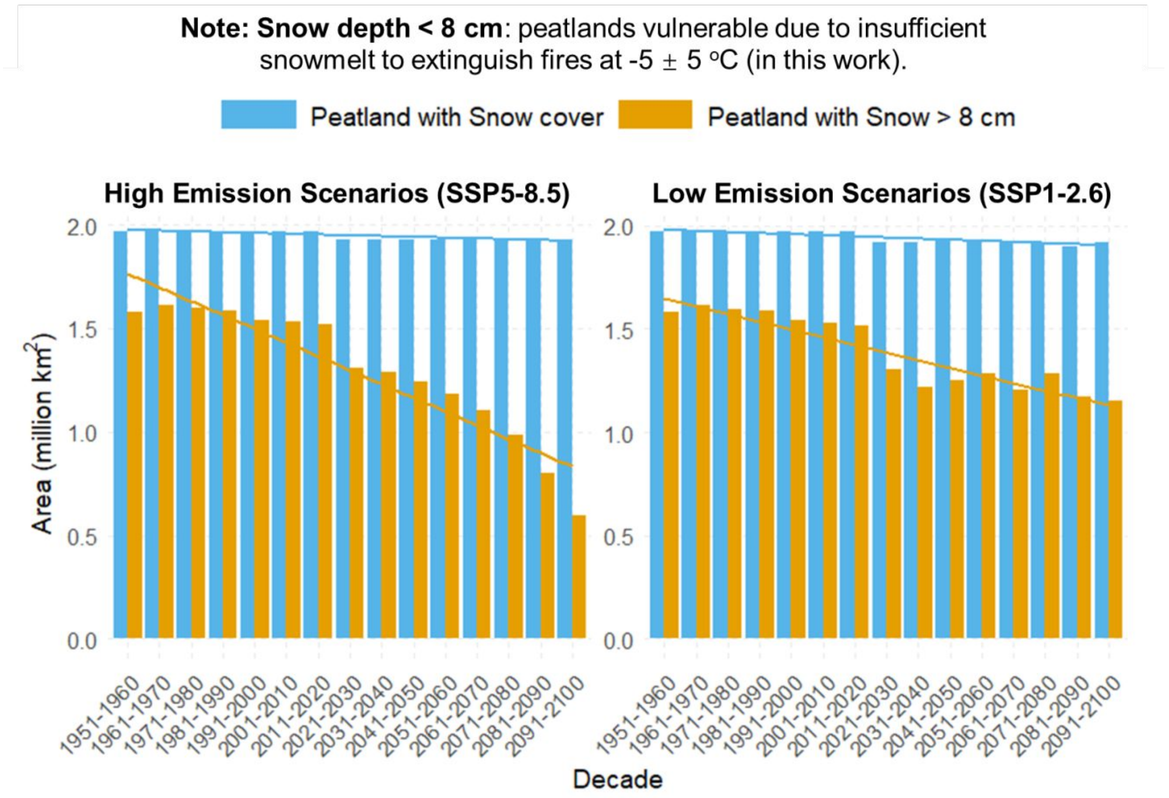

Fig. S4. Historical and projected decade-averaged areas of peatland with snow cover, and those with > 8 cm snow cover. The latter identified in this work as a safe threshold for extinguishing peat fires at -5 ± 5 °C. Data source: PEATMAP, ERA5-Land monthly averaged data from 1950 to 2020, and CMIP6 models under SSP1-2.6 (optimistic scenario) and SSP5-8.5 (pessimistic scenario), using the primary ensemble member (r1i1p1).

Fig. S4 is to estimate the area of vulnerable and safe peatland regions based on the safe snowmelt extinction threshold (minimum snow layer thickness of 8 cm at -5 ± 5 °C) identified in this work. Global peatland distribution data is derived from PEATMAP by Xu et al.[1] Historical snow depth data between 1951-2020 is from ERA5-Land (fifth-generation European Centre for Medium-Range Forecasts reanalysis).[2] Snow depth projections under two Shared Socioeconomic Pathways (SSP) is from the Scenario Model Intercomparison Project for the Coupled Model Intercomparison Project 6 (CMIP6).[3] Both optimistic scenario (SSP1-2.6) and pessimistic scenario (SSP5-8.5) use the primary ensemble member (r1i1p1).

Results show that, from 1951 to 2020, the peatland with snow cover shows variation but no significant decrease is observed ($p = 0.408$). However, The peatland with snow depth > 8 cm has been significantly decreasing ($p = 0.0135$). Notably, both peatland with snow cover and peatland with snow

depth > 8 cm are projected to have a decline till 2100 ($p < 0.001$). Under SSP5-8.5, the pessimistic scenario, the safe peatland area will decrease by 54.3%, while under SSP1-2.6, the optimistic scenario, it will decrease by 11.5%.

References:

(1) Xu, J.; Morris, P. J.; Liu, J.; Holden, J. PEATMAP: Refining Estimates of Global Peatland Distribution Based on a Meta-Analysis. *Catena* **2018**, *160* (September 2017), 134–140. https://doi.org/10.1016/j.catena.2017.09.010.

(2) Muñoz-Sabater, J.; Dutra, E.; Agustí-Panareda, A.; Albergel, C.; Arduini, G.; Balsamo, G.; Boussetta, S.; Choulga, M.; Harrigan, S.; Hersbach, H. ERA5-Land: A State-of-the-Art Global Reanalysis Dataset for Land Applications. *Earth Syst. Sci. data* **2021**, *13* (9), 4349–4383.

(3) O'Neill, B. C.; Tebaldi, C.; Van Vuuren, D. P.; Eyring, V.; Friedlingstein, P.; Hurtt, G.; Knutti, R.; Kriegler, E.; Lamarque, J.-F.; Lowe, J. The Scenario Model Intercomparison Project (ScenarioMIP) for CMIP6. *Geosci. Model Dev.* **2016**, *9* (9), 3461–3482.
